# Phenotyping Fusarium head blight through seed morphology characteristics using RGB imaging

**DOI:** 10.3389/fpls.2022.1010249

**Published:** 2022-10-18

**Authors:** Fernanda Leiva, Mustafa Zakieh, Marwan Alamrani, Rishap Dhakal, Tina Henriksson, Pawan Kumar Singh, Aakash Chawade

**Affiliations:** ^1^ Department of Plant Breeding, Swedish University of Agricultural Sciences, Lomma, Sweden; ^2^ Lantmännen Lantbruk, Svalöv, Sweden; ^3^ International Maize and Wheat Improvement Center (CIMMYT), Texcoco, Mexico

**Keywords:** Fusarium head blight, seed phenotyping, seed morphological characters, wheat, visual scores, SmartGrain, Cgrain Value™

## Abstract

Fusarium head blight (FHB) is an economically important disease affecting wheat and thus poses a major threat to wheat production. Several studies have evaluated the effectiveness of image analysis methods to predict FHB using disease-infected grains; however, few have looked at the final application, considering the relationship between cost and benefit, resolution, and accuracy. The conventional screening of FHB resistance of large-scale samples is still dependent on low-throughput visual inspections. This study aims to compare the performance of two cost–benefit seed image analysis methods, the free software “SmartGrain” and the fully automated commercially available instrument “Cgrain Value™” by assessing 16 seed morphological traits of winter wheat to predict FHB. The analysis was carried out on a seed set of FHB which was visually assessed as to the severity. The dataset is composed of 432 winter wheat genotypes that were greenhouse-inoculated. The predictions from each method, in addition to the predictions combined from the results of both methods, were compared with the disease visual scores. The results showed that Cgrain Value™ had a higher prediction accuracy of *R* ^2^ = 0.52 compared with SmartGrain for which *R* ^2^ = 0.30 for all morphological traits. However, the results combined from both methods showed the greatest prediction performance of *R* ^2^ = 0.58. Additionally, a subpart of the morphological traits, namely, width, length, thickness, and color features, showed a higher correlation with the visual scores compared with the other traits. Overall, both methods were related to the visual scores. This study shows that these affordable imaging methods could be effective to predict FHB in seeds and enable us to distinguish minor differences in seed morphology, which could lead to a precise performance selection of disease-free seeds/grains.

## Introduction

In the countries of the Baltic Sea region, the most widely cultivated crop is winter wheat (*Triticum aestivum L*.), (Shiferaw et al., 2013; Chawade et al., 2018). While efforts are made to achieve sustainable intensification of high grain yields in wheat production, the emergence and increase in the virulence of plant pathogens conversely leave the nutritional integrity and production of wheat grains at risk (Castro Aviles et al., 2020). The decrease in grain quality and protein content negatively impacts the use of the grains and therefore affects food security and safety (Asseng et al., 2019). Fusarium head blight (FHB), mainly caused by the fungus *Fusarium graminearum* Schwabe [teleomorph: *Gibberella zeae* (Schwein) Petch], is one of the wheat diseases with a major impact on wheat grain yield and quality. FHB can dramatically reduce grain quality and yield through the formation of sterile and wizened florets. FHB-infected grains suffer from major marketing, consumption, and processing constraints, which is the buildup of mycotoxins—mainly deoxynivalenol (DON) (Del Ponte et al., 2022). DON inhibits protein synthesis, cutting off normal cell function, which is hazardous for the consumption of humans and animals (Polak-Śliwińska and Paszczyk, 2021). FHB disease management strategies rely on integrating several cultural practices such as fungicide treatment, crop rotation, mixed culture, and tillage (Gilbert and Haber, 2013). However, growing FHB-resistant cultivars is seen as a more sustainable and durable strategy for mitigating disease epidemics, thus avoiding large economic losses. Hence, identifying sources of novel resistance is a key component in pre-breeding activities that can be introgressed to develop commercial FHB-resistant cultivars.

The resistance components for FHB, commonly known as resistance types, have been defined into type I to type V (Mesterhazy, 2020): type I is resistance to initial infection, type II is resistance to disease spread (Schroeder and Christensen, 1963), type III is resistance to damage of Fusarium-damaged kernels (FDK), type IV is resistance to the buildup of DON toxins, and type V is tolerance. Traditionally, studies on FHB resistance have relied on measuring the symptoms in spikes and kernels (resistance types II and III). Type II is assessed by rating the visual symptoms on the spikes, which appear as bleached, yellowish or discolored, and stunted (Zakieh et al., 2021; Steed et al., 2022). FDK is quantified traditionally by estimating the amount of visibly damaged kernels, which appear smaller, shriveled, and in a range of colors from pale pink to brown (Delwiche et al., 2010), according to a predetermined scale for visual assessments or by employing manual tools (Ackerman et al., 2022). Comparisons between both types of resistance (resistance types II and III) have revealed that it would be more efficient and consistent to estimate FHB than the degree of colonization on the spike (Agostinelli, 2009; Balut et al., 2013; Khaeim et al., 2019; Ackerman et al., 2022). However, screening by either manual or visual assessments is a labor- and time-consuming process for rating genotypes, is biased due to the subjectivity of visual assessments, and has low reproducibility among experiments (Barbedo et al., 2015; Khaeim et al., 2019). As a result of the previously cited limitations, the use of image analysis approaches has been investigated to evaluate FDK, particularly in estimating morphological characteristics. However, the existing different imaging approaches have their disadvantages and trade-off in terms of costs, time expenses, resolution, and precision when considering an application (Saccon et al., 2017).

Among the investigated methods, Iwata and Ukai (2002) and Iwata et al. (2010) investigated changes in grain shape using elliptic Fourier descriptors of two- and three-dimensional features from vertically and horizontally located seed images. Despite the accuracy reached, there are limitations in terms of image resolution and regarding the manual handling of samples during the procedure. Menesatti et al. (2009) presented a method to classify FHB in wheat-infected kernels—according to the shape criteria—into the following groups: chalky, shriveled, or healthy. The method proved to be functional to categorize kernels as chalky or healthy, but not for shriveled or gravely affected samples. Jirsa and Polišenská (2011) developed a model for the identification of Fusarium-damaged wheat kernels using image analysis. The characterization of healthy or damaged kernels based on color parameters revealed a high accuracy compared with the shape and DON content parameters. However, image processing was done with manual selections and comparing only 40 kernels—either heavily damaged or healthy—without considering any halfway stage. Similarly, the use of hyperspectral imaging for detecting Fusarium sp. in seeds has been previously investigated (Delwiche et al., 2010; Shahin and Symons, 2011; Bauriegel and Herppich, 2014; Barbedo et al., 2015; Femenias et al., 2022; Rangarajan et al., 2022; Yipeng et al., 2022). The methods have been shown to be accurate and have identified more factors involved in FDK. A more advanced technique based on X-ray computed tomography has been implemented for evaluating seed shape in finer detail (Gomes and Duijn, 2017; Liu et al., 2020). Nevertheless, inconsistencies because of specular reflection, correct wavelength selection, kernel orientation, selection of reference parameter, costs of acquisition devices, and the storage requirement for highly dimensional and massive data sets may be limiting the application of these methods (Dissing et al., 2013; Lu et al., 2020).

In the face of the constraints cited earlier, automated and light-weight free software for grain image analysis have been developed (Wang et al., 2009; Komyshev et al., 2017; Colmer et al., 2020; Zhu et al., 2021); some examples of them are GrainScan (Whan et al., 2014), which analyzes size and color features, and SmartGrain (Tanabata et al., 2012), which analyzes size and shape features. Both software are instantaneous in image recognition despite the position, overlapping, or the number of seeds. Alternatively, commercially available imaging instruments for grain image analysis combine hardware and software, including WinSEEDLE (Regent Instruments Inc.), Seed Count (Next Instrument Pty Ltd.), Vibe QM3 Grain Analyzer (VIBE), and Cgrain Value™ (Cgrain AB). The instruments use optical or flatbed scanners to extract features such as size, shape, and color in the color representation hue, saturation, and light (HSL). However, SeedCount and Vibe QM3 Grain Analyzer only scan the top surface of the samples, thus omitting morphological characteristics that are not in the viewing area. A more advanced instrument is Videometer Lab (Videometer A/S, Denmark), which provides rapid color, shape, and texture measurements. Videometer Lab is ideal to use in analyzing kernel surfaces, but it requires certain expertise and allows the analysis of only a few samples at once.

In this context, this paper has three objectives; first is to investigate the applicability of low-cost digital image analysis to predict FHB infection in harvested grains through morphological traits. This will offer more insight into the traits that are correlated to the degree of FDK. The second objective is to compare the applicability of the two methods used for grain image analysis—SmartGrain, and Cgrain Value™—in terms of consistency and throughput. The third one is to illustrate the processing chain and result interpretation with a descriptive data analysis.

## Materials and methods

### Plant material

Wheat kernel samples were collected from an experiment under accelerated indoor growth conditions (Zakieh et al., 2021) using winter wheat genotypes from two different sources. The first source consisted of 338 genotypes (breeding set) provided by the Swedish agricultural cooperative (Lantmännen Lantbruk, Svalöv, Sweden). The second source consisted of 181 germplasm genotypes (genebank set) provided by the Nordic Genetic Resource Center (Nordgen), with highly diverse plant materials including landraces and old cultivars.

### Experimental design/growth and inoculation protocol

Plants were grown following an augmented block design in a climate-controlled chamber. After germination, the plants were subjected to a vernalization period of 57 days at 3°C with 8 h of daily light at medium–high light intensity (LI) of 250 μmol m^−2^ s^−1^. At the end of the vernalization period, the climatic conditions were adjusted with a gradual increase in temperature and LI for the acclimatization of the plants to the next phase of accelerated growth conditions. Once the acclimatization period was concluded, the plants were left to grow at a constant temperature of 22°C. The accelerated growth conditions were adapted by exposing the plants to a prolonged daily light duration of 22 h, with LI at 400 μmol m^−2^ s^−1^ of uniform light intensity from LED light plates. Under these accelerated growth conditions, the plants were watered daily and fertilized weekly using first a combination of a high-phosphate and high-nitrogen soluble fertilizer SW-BOUYANT 7-1-5 + Mikro + KH_2_PO_4_, then only with a high-nitrogen fertilizer, and finally with a high-potassium soluble fertilizer Yara Tera Kristalon NPK 12-5-30 with S and Mikro.

After completing the anthesis stage, at 33 days post-acclimatization, the plants were moved to a glasshouse chamber with relative humidity (rh) of 60% and a constant temperature of 24°C for 24 h to allow their adaptation to the new growth conditions prior to inoculation. Thereafter, the winter wheat spikes were spray-inoculated with an inoculum suspension prepared from the harvested spore of *F. graminearum* and *F. culmorum*, with a concentration of 5 × 10^5^ spore/ml. Subsequently, the plants were left to incubate at 90% rh with 16/8 h dark/light cycle at a constant temperature of 24°C for 48 h before adjusting the climatic conditions back to 60% rh. The plants were eventually left to grow under the latter conditions for 24 days before harvesting the seeds. Eight isolates from *F. graminearum* and *F. culmorum* species were used in inoculating the plants provided by the Swedish agricultural cooperative Lantmännen Lantbruk. An inoculum preparation was carried out by incubating the fungal spores at 24°C for 4 days in dark conditions to allow for mycelial growth on SNA media plates. Later, the fungal plates were exposed to near ultra-violet UV radiation for 10 h to induce macroconidia formation. Afterward, the fungal plates were incubated for 4 days at 24°C in dark conditions. Finally, macroconidia spores were collected to make the inoculation suspension with the provided concentration after adding the surfactant Tween^®^20 0.002% (v/v) final volume of the inoculum. A more detailed protocol is described in Zakieh et al. (2021).

### FHB visual assessment

In order to evaluate FHB resistance on a large number of genotypes, a modified visual scoring of the FHB disease severity method was adopted. The method took into account the incidence of all FHB symptoms across the main tiller spike of each genotype. Therefore, disease severity was assessed as the percentage score of infected spikelets relative to all spikes, regardless of symptom continuity on the same spike. FHB development was scored at 6, 8, 10, and 12-days post-inoculation (dpi) (Stack and McMullen, 1998). The FHB disease severity scores varied between 100 to 5% for the most susceptible phenotypes and the most resistant ones, respectively. Finally, the results of the visual scores were validated by association mapping, thus identifying the quantitative trait loci of FHB resistance.

### Seed shape parameters

Two different grain phenotyping methods were employed in this study: an automated imaging instrument with software and hardware named Cgrain Value™ which is commercially available (Cgrain AB) and the free software named SmartGrain developed by Tanabata et al. (2012) and can be downloaded from the Quantitative Plant website (Lobet, 2017). The implementation of both methods is described in the following sections.

### SmartGrain

**Figure 1:**
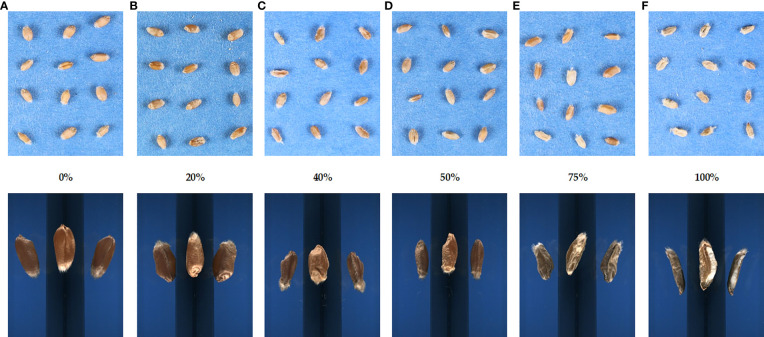
Images of the different levels of Fusarium head blight severity on winter wheat seeds. The rating of disease severity ranged from (**A**) 0 to (**F**) 100%. Scoring was based on the proportion of total infected spikes to the total amount of spikes. The top images were obtained for the SmartGrain analysis, and the bottom images were acquired using the Cgrain Value™ instrument.

For image acquisition, the seeds were captured with a low-cost image protocol acquisition from a top-view angle of 55 cm above the seeds and placed manually on a flat surface using a digital single-lens reflex camera Canon EOS 1300D (Canon U.S.A. Inc., Huntington, NY, USA), which has a resolution of 18 megapixels, mounted on a Kaiser RS-1 repro stand. The camera was tethered to the software digiCamControl (Istvan, 2014) with optimal exposure settings based on the best seed view, F-Stop 1/160, exposure time 1/10, and ISO 800. The seeds were placed manually per genotype uniformly on a blue cardboard that was used as a background on a stand aside from a 15-cm ruler for further analysis. Digital images were stored with 3,456 × 2,304-pixel resolution in JPEG format (**Figure 1**, top images).

The image analysis was thereafter carried out using SmartGrain software following its default protocol (Tanabata et al., 2012). Briefly, the image scale was set up by taking a known sample from the ruler and registering it on the software. Then, the segmentation method by color was chosen, the precision sensibility was set at the minimum value of “1”, and the seed detection intensity was at a maximum value of “4” to obtain all possible shape details; the rest of the parameters were set to default. Finally, all the processed images were saved as TIFF files, and the results were saved in a CSV format. The software provides seven morphological characteristics: area seed (AS), perimeter length (PL), length (L), width (W), length-to-width ratio (LWR), circularity of the seed (CS), distance between the intersection of length and width, and the center of gravity (DS). **AS** corresponds to the total number of pixels of the segmented seed, this parameter estimates the seed size. **PL** refers to the length measurement of the seed outline. **L** corresponds to the major length measurement in the axis and **W** to the minor length axis measurement. **CS** estimates how round the region of interest is (seed), and it is calculated as $\frac{4\times \pi \times AS}{PL^{2}}$ . **LWR** is calculated by $\frac{L}{W}$ , and it provides an idea of the seed shape between rectangular and circular depending on the value. The distance between the transverse axis from the outline of the seed (IS) and the center of gravity (CG) is used to estimate **DS** [described in detail by Tanabata et al. (2012)].

### Cgrain Value™

For single kernel analysis, seeds were scanned with Cgrain Value™, which is an analytical imaging instrument. The device inspects each kernel through a unique mirror design covering more than 90% of the grains’ surfaces in every image. The analysis starts by pouring into the metal bowl of the Cgrain Value™ a batch of seeds per line and per genotype. The seeds rotate into the bowl and then, one by one, are photographed and analyzed simultaneously. After the analysis is completed, three different reports are created (result file, stat file, and image file). The result file consists of the morphological characteristics for each batch of seeds (seed count, thousand kernels, *etc*.), the stat file provides data per individual seed of a group (length, width, *etc*.), and the image file corresponds to the single seed images acquired (**Figure 1**, bottom images).

The instrument provides nine morphological attributes: length (L), width (W), thickness (T), average width (AVG.W), volume (V), weight (WT), light, hue, and saturation. Parameters such as L, W, and T are estimated by taking the longitudinal measurement of the axis major, higher minor, and minor, respectively. In the case of AVG.W, as the seed is received as a three-dimensional image, the measurement is referring to the mean of the average curvature. V corresponds to the seed volume obtained from the 3D image. For WT, the device has an internal balance, so while acquiring the image, it also weighs the grain. Color parameters, hue, saturation, and light are also determined by the instrument; it specifies the color base of a sample, how saturated it is, and how bright it is, respectively.

### Statistical analysis

Statistical analyses were conducted using R (Team, R. C, 2013). The visual scorings of the last time-point on infected spikes, including cultivars with zero symptoms, were included in a file together with the mean values per genotype of the results given by Cgrain Value™ and SmartGrain. Each replicate of the data set was filtered by missing data (NA). Those with NA along the four replicates were removed and those with presence in more than one replicate were substituted using FactoMineR (Lê et al., 2008) and missMDA (Josse and Husson, 2016) packages. Then, using the Agricolae R package (De Mendiburu, 2014), the checks in each augmented block were used to adjust the means for each trait per replicate, the model of which is as follows:


$$y_{il}=u+G_{il}+\beta_{1}+\epsilon_{il}$$

where *y_il_* corresponds to the adjusted means of the *i* ^th^ wheat cultivar in the *l* ^th^ block, *u* is the general mean value, *G_il_* is the effect of the *i* ^th^ wheat genotype in the *l* ^th^ block, *β*_1_ is the *l* ^th^ block effect, and *ϵ*_*il*_ is the residual. Subsequently, using the adjusted means, the best linear unbiased estimates (BLUEs) was calculated using the randomized complete block design option in META-R 6.04 (Alvarado et al., 2015) based on the following model:


$$y_{ijm}=u+S_{j}+G_{ijm}+R_{m}+\epsilon_{ijm}$$

where *y_ijm_* corresponds to the BLUE of the *i* ^th^ genotype from the *j* ^th^ population in the *m* ^th^ replicate, *u* is the general mean value, *S_j_* is the effect of the *j* ^th^ source of material, *G_ijm_* is the effect of the *i* ^th^ genotype in the *m* ^th^ replicate, *R_m_* is the *m* ^th^ replicate of the effect, and *ϵ*_*ijm*_ is the residual effect. The source of wheat genotypes *S_j_* was considered the grouping factor.

The BLUEs data previously centered were used to predict FHB using a multiple regression model:


$$y_{i}=\beta_{0}+\beta_{1}x_{i1}+\beta_{2}x_{i2}+\ldots +\beta_{p}x_{ip}+\epsilon$$

Where for i=n observations: *y_i_* corresponds to the dependent variable, *x_i_* to the explanatory variables, *β*_0_ corresponds to y-intercept (constant term), *β_p_* corresponds to the slope coefficients for each explanatory variable, and ϵ corresponds to the error of the model (also known as the residuals). Three models were created using the morphological traits provided by both methods (Cgrain Value™ and SmartGrain) as independent variables and visual scorings as the dependent variable. One model combines all the traits, and two others use the traits provided by each method. To build each model, the data set was partitioned employing the function “createDataPartition” of the caret package (Kuhn et al., 2020) into 70% for model training (training set) and the remaining 30% for evaluating model performance (test set). Subsequently, the model was fitted to the training set, and it predicted the responses using the test set. To evaluate the quality of the predictions and mitigate the possibility of errors due to the random data partitioning, the cross-validation was executed 100 times, which means resampling the data set, and the mean of the criterion was taken as the final result.

## Results

**Figure 2:**
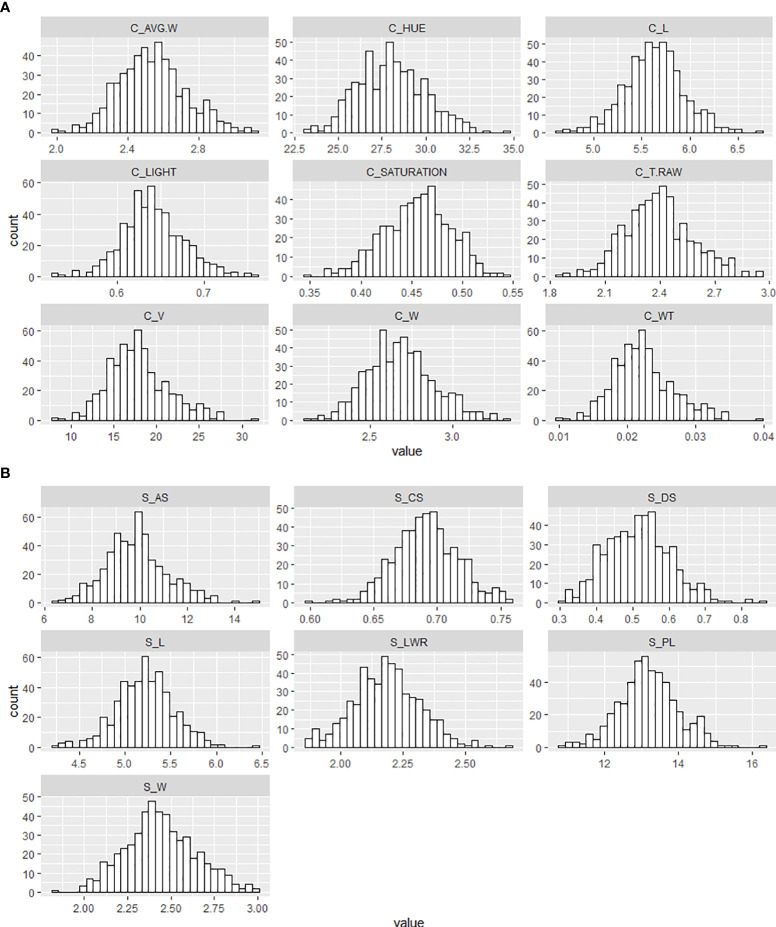
Frequency distribution of the different morphological traits of wheat genotypes seeds from the breeding and genebank sets collected with **(A)** the Cgrain Value™ instrument and **(B)** the SmartGrain software.

This study examined a total of 16 morphological traits, including size, color, and shape of winter wheat grains from the genebank and breeding sets with different levels of FHB infection. Nine traits were obtained with the instrument Cgrain Value™ and seven traits with the software SmarGrain. The distribution of all the morphological traits measured by the two methods showed a Gaussian distribution (**Figure 2**). In order to understand the association between these traits and FHB resistance, a comparison with the traits measured of 80 FHB susceptible and resistant genotypes was performed. For this purpose, five genotypes per replicate (four replicates) from both sets, breeding and genebank, were selected based on the FHB severity scores on the spikes, genotypes scored as 0% (visually non-infected or resistant), and ones scored as 100% (visually infected or susceptible). Among the infected and non-infected selected groups, there was a 22.61% reduction in V and 11.32% in AS. Other parameters also showed a reduction, such as T_RAW at 10.60%, W at 8.30% in both methods, and WT at 22.63%. Additionally, L was reduced according to the results by 1.96% in Cgrain Value™ and 2.26% in SmartGrain. Similarly, CS and PL showed a decrease, but in less proportions with 4.60 and 3.25%, respectively. The minimum seed L measured was 4.59 mm for non-infected and 4.50 mm for infected genotypes. On the other hand, color parameters expressed major changes compared with all the other morphological traits. Hue and the light increased with the infection by 19.91 and 8.28%, respectively, while saturation decreased at about 15.52% (**Table 1**). According to the analysis of variance (two-way ANOVA), the morphological traits L, W, T_RAW, light, and hue were highly significant (*P*< 0.001), likewise with V, CS, and saturation (*P*< 0.01), indicating a clear association with FHB disease severity level. Meanwhile, the parameters WT, AS, LWR, PL, and DS did not indicate any significance but still showed slight differences between infected and non-infected grains.

**Table 1:** Descriptive statistics showing differences between the seed shape characters of 80 genotypes from genebank and breeding set under non-infection (0%) and full infection (100%) FHB symptoms, with five genotypes of each one per replicate.

a) CGRAIN VALUE™										
Description	Level	L	W	T.RAW	AVG.W	V	WT	HUE	SAT	LIGHT
Mean	**Non_Infected**	5.6	2.76	2.47	2.61	19.18	0.02	25.78	0.48	0.62
	**Infected**	5.49	2.53	2.2	2.36	14.84	0.01	30.81	0.4	0.68
% Reduction		1.96	8.29	10.6	9.41	22.61	25	-19.51	16.52	-9.67
Max	**Non_Infected**	6.88	3.7	3.245	3.41	38.9	0.04	30.46	0.55	0.715
	**Infected**	6.46	3.13	2.93	3.03	26.6	0.03	38.99	0.51	0.81
Min	**Non_Infected**	4.59	2.18	1.98	2.08	10.66	0.01	23.45	0.43	0.55
	**Infected**	4.5	2.05	1.88	1.96	7.1	0.008	24.88	0.3	0.58
SD	**Non_Infected**	0.52	0.36	0.3	0.32	6.74	0.008	1.32	0.02	0.04
	**Infected**	0.45	0.23	0.22	0.23	4.02	0.005	3.01	0.05	0.05
SE	**Non_Infected**	0.08	0.05	0.048	0.05	1.06	0.001	0.21	0.004	0.006
	**Infected**	0.07	0.04	0.036	0.04	0.63	0.0007	0.47	0.008	0.007
CV (%)		9.44	13.02	12.26	12.53	35.15	35.15	5.14	5.8	6.79
b) SMARTGRAIN										
Description	Level	AS	PL	L	W	LWR	CS		DS	
Mean	**Non_Infected**	9.77	12.91	5.08	2.44	2.13	0.7		0.48	
	**Infected**	8.66	12.49	4.97	2.23	2.25	0.67		0.51	
% Reduction		11.32	3.25	2.26	8.27	-5.64	4.6		-6.9	
Max	**Non_Infected**	17.36	17.15	6.57	3.71	2.53	0.8		0.85	
	**Infected**	13.63	15.54	6.25	2.95	2.65	0.73		1.01	
Min	**Non_Infected**	3.41	7.91	3.2	1.39	1.53	0.63		0.24	
	**Infected**	3.01	7.31	2.88	1.36	1.88	0.61		0.23	
SD	**Non_Infected**	3.21	2.16	0.81	0.48	0.17	0.03		0.13	
	**Infected**	2.55	1.95	0.79	0.38	0.15	0.02		0.18	
SE	**Non_Infected**	0.5	0.34	0.12	0.07	0.02	0.005		0.02	
	**Infected**	0.4	0.3	0.12	0.06	0.02	0.004		0.02	
CV (%)		32.85	16.72	16.11	19.72	8.41	4.76		28.27	

a) Cgrain Value™ size, shape and color characteristics, (L) [mm], Width (W) [mm], Raw Thickness (T.RAW) [mm], Mean Width (AVG.W) [mm], Weight (WT) [g], Hue, Saturation, and Light; b) SmartGrain size and shape characteristics, Area size (AS) [ mm2], Perimeter length (PL) [mm], Length (L) [mm], Width (W) [mm], Length to width ratio (LWR), Circularity (CS) Distance between IS and CG (DS) [mm].

**Figure 3:**
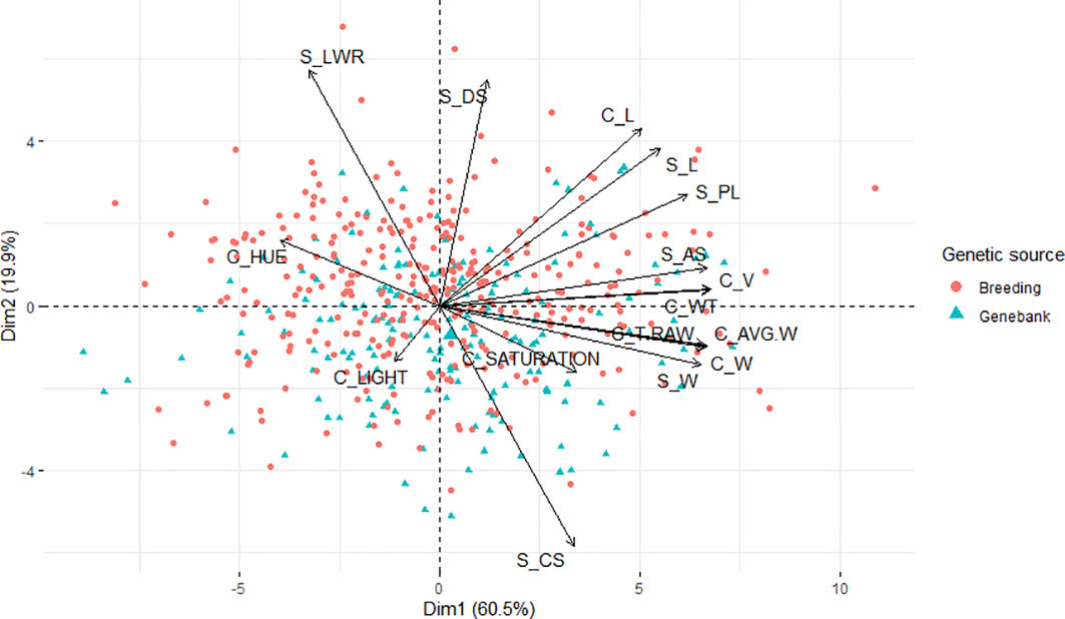
Principal component analysis biplot of the morphological traits collected with Cgrain Value™ and SmartGrain of the breeding and genebank seeds infected with different levels of Fusarium head blight.

Additionally, a principal component analysis (**Figure 3**) was performed to show the response of all the seed traits studied regarding the disease infection and how they correlate to each other. The proportion of total variance on the two first principal components and correlations represents 60.50 and 19.90%, respectively, of the total variance. The LWR trait was shown to be the higher positive in the first principal component; similarly, hue was shown to be positive but in a lesser proportion. In the same component but with negative loading, we found CS as the variable with the highest contribution; the traits W from both methods, AVG.W, and T_RAW were also projected onto this component with a loading of a slightly lesser norm. Although saturation was also projected onto this component, it was shown to be the smallest loading. On the other hand, in the second principal component, the traits DS and L from both methods, PL, AS, V, and WT showed a high positive loading with similar proportions, whereas the trait light was the only one with a negative loading into the second principal component and the one with less projection among all the traits. In general, all the seed morphological traits assessed expressed variability and influence in the two principal components. In addition, as can be observed in the graph, the variation of LWR has an opposite projection to the CS trait, expressing a good indicator to study the deformation of the grains caused by the disease infection.

**Figure 4:**
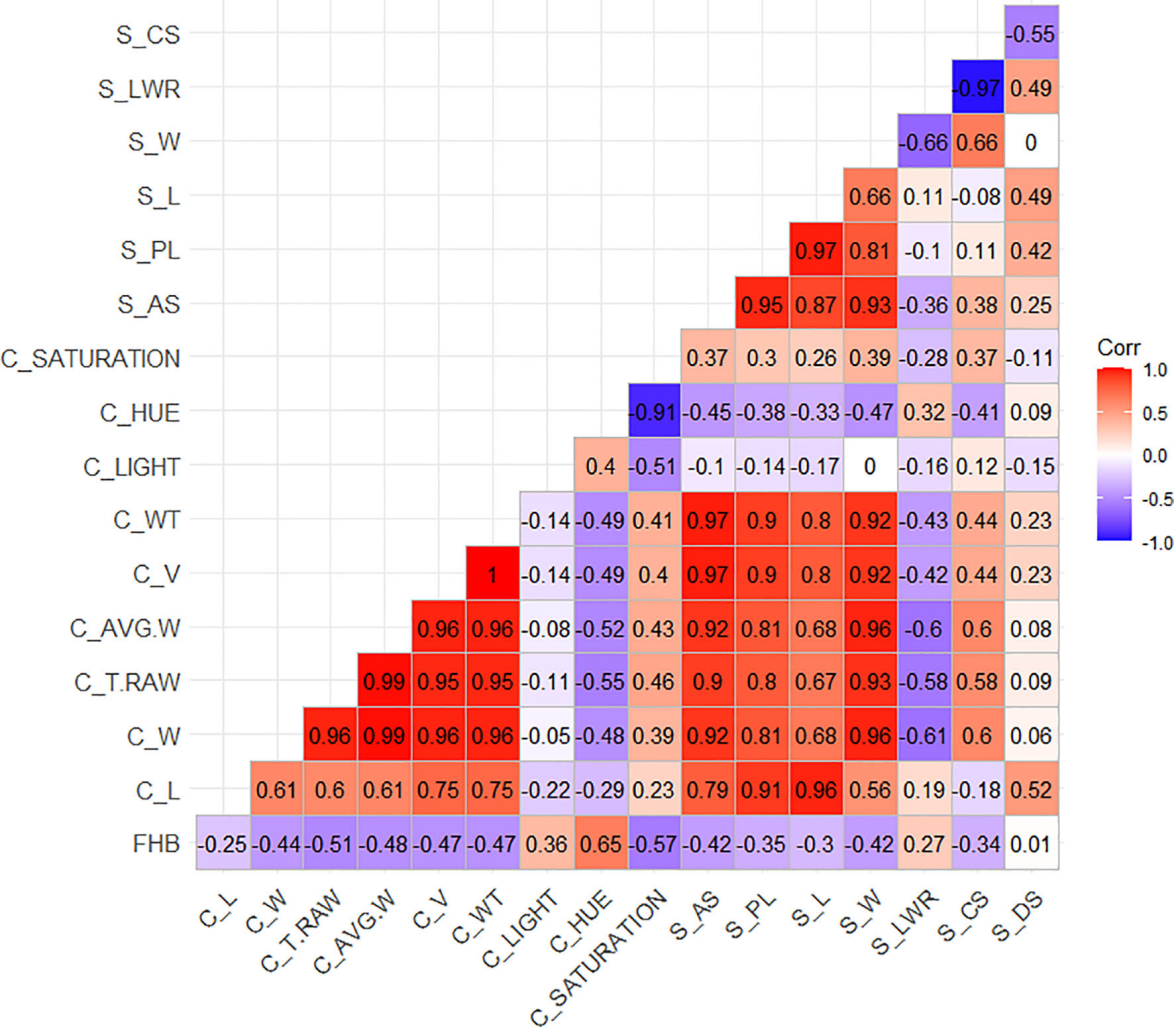
Sorted upper triangle correlation matrix among the morphological attributes of the wheat genotype seeds from the breeding and genebank sets collected with the Cgrain Value™ and the SmartGrain software.

Considering **Table 1**, the mean values for the same morphological traits measured by both methods (L and W) across the two sets, genebank and breeding, were similar. The difference between infected and non-infected seeds was 0.11 mm in L in both methods and between 0.21 and 0.25 mm in W and AVG_W. Both methods provide important parameters for seed morphology studies. Cgrain Value™ provides V and WT values and color information. Although these are important characteristics for different study purposes, mainly for identifying FHB-infected kernels, SmartGrain, in turn, provides information such as PL, AS, and CS that can show variabilities between infected and non-infected seeds. Here the BLUES for all the measured parameters were correlated with each other and in association with the visual scorings on the spikes (**Figure 4**). A moderate to high positive correlation was found with the color parameter hue, and a low positive correlation with light was given by Cgrain Value™ and LWR as well as given by SmartGrain (*r* = 0.65, *r* = 0.36, and *r* = 0.27, respectively). Negative correlations were also found between the visual evaluations of symptoms and the other characteristics in different levels of strength of association. There was no correlation between FHB visual scoring and DS (*r* = 0.01).

**Figure 5:**
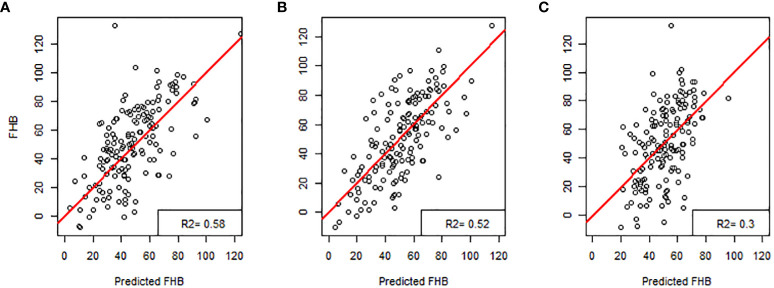
Regression models for predicting Fusarium head blight in wheat: **(A)** all the characteristics obtained with Cgrain Value™ and SmartGrain, **(B)** Cgrain Value™ morphological traits, and **(C)** SmartGrain morphological traits.

**Figure 6:**
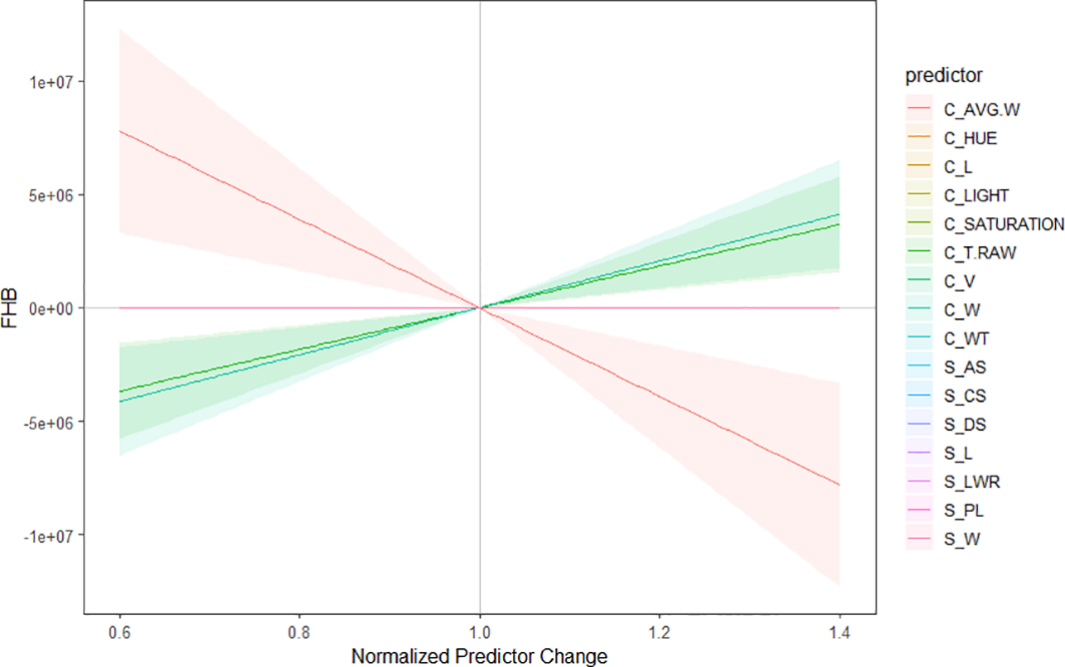
Sensitivity plot of the morphological characteristics to predict Fusarium head blight in wheat. The parameters are organized from the best predictors to the less significant to predict the disease. Color lines indicate the significance, considering red as the most important predictor and pink as the less important one. The highlighted regions reflect the correlation of the parameters among each other.

The multiple linear regression model developed to identify the contributions of the 16 different morphological traits provided by Cgrain Value™ and SmartGrain expressed a high moderate prediction (*R* ^2^ = 0.58), (**Figure 5A**). Aiming to identify which of both methods used in this study provides a higher prediction and also to identify the best morphological traits to predict FHB, two more models were constructed: one for the results given by Cgrain Value™ and another one for the results of SmartGrain. The model of Cgrain Value™ traits showed a moderate prediction (*R* ^2^ = 0.52), (**Figure 5B**). On the other hand, the model of SmartGrain traits showed medium–low prediction (*R* ^2^ = 0.30), (**Figure 5C**), clearly showing that the first model had a higher prediction than separately. In addition, the morphological parameters that are the most suitable to assess FHB in grains above all the 16 evaluated were identified. According to the regression model and the ANOVA analysis, the parameters that provided more information about the disease are the length, width, thickness, average width, circularity, and the color parameters in the color representation HSL (**Table 2**). The sensitivity test showed that these variables provide the highest value of *R*-square, (*R* ^2^ = 0.52). These morphological traits are enumerated from most significant to least significant in **Figure 6**.

**Table 2:** Summary of the multiple linear regression model combining all the 16 morphological characteristics provided by Cgrain Value™ and SmartGrain.

Model summary					
Morphological traits	Sum sq	Mean sq	*F*-value	Pr (>*F*)	
C_L	23,829	23,829	64.587	6.99E-15	***
C_W	51,079	51,079	138.446	< 2e-16	***
C_T.RAW	40,500	40,500	109.772	< 2e-16	***
C_AVG.W	2,013	2,013	5.456	0.0199	*
C_V	2,603	2,603	7.055	0.00816	**
C_WT	680	680	1.843	0.17526	
C_LIGHT	31,656	31,656	85.802	< 2e-16	***
C_HUE	39,386	39,386	106.752	< 2e-16	***
C_SATURATION	2,649	2,649	7.18	0.00762	**
S_AS	178	178	0.483	0.48734	
S_PL	624	624	1.691	0.1941	
S_L	3,027	3,027	8.204	0.00436	**
S_W	45	45	0.121	0.72828	
S_LWR	0	0	0.001	0.9802	
S_CS	1,651	1,651	4.476	0.03489	*
S_DS	539	539	1.461	0.22731	

The most significant characteristics concerning the Fusarium head blight disease infection according to the P-value has an *. (No significance P>0.05; *P≤0.05; **P≤0.01; ***P≤0.001).

## Discussion

This study compared the potential performances of two different image-based methods to predict FHB. The results of both indicated that morphological seed traits are functional for predicting FHB among two different sets of genotypes evaluated. Furthermore, a comparison of the applicability of the two methods was properly addressed by evaluating the cost, accuracy, and time efficiency—for instance, to extract dimension, shape, and color parameters, Cgrain Value™ utilizes a unique mirror design to inspect all possible angles of individual kernels in the sample. Additionally, image capture and processing are instantaneous, thanks to the hardware and software combination. Conversely, image acquisition using the SmartGrain system was carried out over a relatively long period, yet image processing was done relatively fast. However, compared with Cgrain Value™, the earlier approach is cheaper considering the cost of the tools used in image capture, requiring a simple RGB camera, a static frame, and the free software.

On the other hand, the morphological traits, based on the statistical analysis results, that showed significant correlations to the visual scores were color traits in the HSL color representation and thickness from Cgrain Value™, length and width, from both methods (**Figures 5**, **6**). Although the other measured morphological traits were not significantly correlated to the visual scores, infected grains still expressed differences in these traits that may be ultimately informative about seed health and refine the prediction (**Table 1**). Nevertheless, DS was not correlated and did not express significant differences in infected seeds of FHB, but it could prove useful in other applications.

The evaluated visual scores of the symptoms associated with FHB—bleached, yellowish or discolored, and stunted spikes—were previously validated by the identification of several loci by genome-wide association studies (GWAS) in a previous study with the same plants and visual scorings (Zakieh et al., 2021). The proposed methods aim to replace costly and labor-intensive genetic analysis.

Therefore, the prediction of both methods studied here appears to be consistent for FHB with the assigned traits concerning the phenotype–genotype association. Previous investigations showed a high correlation between symptoms that are present on wheat heads and the rate of kernel damage (Góral et al., 2018). Therefore, it is feasible to reference the estimated visual scores of disease severity to establish similar results of association/disassociation with the corresponding assessments of grain traits following the methodology in this study.

An important aspect to highlight is that the percentage of disease severity can be assessed, where, in contrast to disease spread from the point of inoculation, it offers less intensive labor by spray inoculation of a larger number of wheat genotypes. Additionally, unlike point-inoculated wheat spikelets, spray-inoculated spikes allow for evaluating the degree of damage caused by the disease to all kernels of the infected spike. Within this work frame, whole spike kernels are investigated for their characteristics rather than the damage to a limited number of kernels caused by Fusarium colonization from the point of inoculation. This, in turn, is expected to shorten the period for disease resistance assessment, lower its cost, and be less labor demanding.

## Conclusion

The results indicated that the traits with a higher correlation to FHB were length, width, thickness, and especially color values in HSL color representation. Moreover, Cgrain Value™ was advantageous to SmartGrain in terms of the time required for image capture and outperformed the latter when applied to a large number of samples, yet SmartGrain processes samples fast and is cheaper in comparison to Cgrain Value™. Although the disease prediction showed a low–moderate accuracy for SmartGrain and a high–moderate accuracy for Cgrain Value™ and the results of both methods combined, this is attributed to the prediction reference, which corresponds to FHB disease severity scorings done on the spikes. However, the novelty of this study resides in the accuracy reached even with a different reference source, but which is directly related. Additionally, as the plant material genotypes and visual scores were validated by GWAS analysis, then the results presented here are phenotype–genotype-associated.

## Data availability statement

The original contributions presented in the study are included in the article/supplementary material. Further inquiries can be directed to the corresponding author.

## Author contributions

AC conceived the study. TH developed the breeding population set. MZ provided the material and the scores of the disease severity. MA performed the image and data acquisition with SmartGrain. RD performed the image and data acquisition with Cgrain Value™. FL analyzed the data and wrote the draft. All authors contributed to the article and approved the submitted version.

## Funding

This study was supported by funding from the SLU Grogrund (SLU.ltv.2019.1.1.1-623), Nordic Council of Ministers (PPP #6P3), and NordForsk (#84597). Formas (#2020-01828).

## Conflict of interest

The authors declare that the research was conducted in the absence of any commercial or financial relationships that could be construed as a potential conflict of interest.

## Publisher’s note

All claims expressed in this article are solely those of the authors and do not necessarily represent those of their affiliated organizations, or those of the publisher, the editors and the reviewers. Any product that may be evaluated in this article, or claim that may be made by its manufacturer, is not guaranteed or endorsed by the publisher.

## Appendix 1

**Table:** Quantitative trait loci (QTL) detected in genome-wide association studies employing seven models at *p* = 0.0001 (LOD ≥ 4) for Fusarium head blight severity in winter wheat from the breeding, genebank, and combined sets (Zakieh et al., 2021). Chr., chromosome; FAF, favorable allele frequencies. The asterisk means also detected by these models at *p* = 0.0002. A, detected above Bonferroni corrected threshold (*α* = 0.05). *B*, the marker effects are estimated for only GLM, MLM, and CMLM and FarmCPU in GAPIT (Lipka et al., 2012).

QTL	Marker	Chr.	Position (cM)	FAF	Effect	Model(s)	Set
SLUfhbchr1B.1	BS00021877_51	1B	154.58	0.06	NA	Blink	Combined
SLUfhbchr2A.2	BobWhite_c16923_64	2A	125.33	0.06	NA	Blink; (SUPER)*	Combined
SLUfhbchr3A.3	Kukri_rep_c89183_282	3A	15.05	0.64	27.84 to 28.10	GLM, CMLM	Combined
SLUfhbchr3B.4	wsnp_Ex_c34975_43204180	3B	67.45	0.95(CS), 0.94 (BS), 0.97(GS)	65.78 to 82.47	GLM, MLM, CMLM, SUPER, MLMM, FarmCPU, Blink	All
	Kukri_c18009_398a	3B	67.67	0.95	78.20 to 80.15	GLM, MLM, CMLM, SUPER	Combined
	wsnp_Ex_c5378_9505533	3B	68.71	0.94	NA	SUPER	Combined
SLUfhbchr3D.5a	RFL_Contig4591_1759	3D	0.00	0.94	51.94 to 54.69*	MLMM; (GLM, MLM, CLM, SUPER, Blink)*	Combined
	RAC875_rep_c115090_5	3D	0.00	0.02	NA	Blink	Breeding
SLUfhbchr3D.5b	JD_c7714_954	3D	143.01	0.04	NA	Blink, SUPER	Genebank
SLUfhbchr5A.6	RAC875_rep_c106118_339	5A	39.02	0.03	-31.55 to -29.40	GLM, MLM, SUPER, MLMM	Combined
SLUfhbch6A.7	Tdurum_contig46670_911	6A	128.26	0.96	NA	SUPER	Combined
SLUfhbchr7A.8	Kukri_c11530_92	7A	232.11	0.84	44.1	CMLM, SUPER, MLMM	Combined
	RAC875_c12733_1509a	7A	228.37	0.83	40.41 to 45.14	GLM, MLM, CMLM, SUPER, MLMM, FarmCPU, Blink	Combined
SLUfhbchr7B.9	wsnp_Ex_c351_689415	7B	143.23	0.02	NA	Blink, SUPER	Breeding
	RAC875_c8752_1079	7B	158.98	0.84	39.97*	SUPER; (CMLM)*	Combined
